# Wernicke’s encephalopathy following radical gastrectomy and prolonged parenteral nutrition: a case report of pyloric obstruction and undisclosed psychiatric comorbidity

**DOI:** 10.3389/fonc.2025.1595004

**Published:** 2025-06-23

**Authors:** Hanchen Ma, Zheng Zhao, Wenjie Zhang, Shuaichen Jin, Linchuan Li, Ruizhao Zong, Xueyu Fu, Yingjie Sun, Yunmiao Pan, Jiankang Zhu, Guangyong Zhang

**Affiliations:** ^1^ Department of General Surgery, Shandong Provincial Qianfoshan Hospital, Cheeloo College of Medicine, Shandong University, Jinan, China; ^2^ Laboratory of Metabolism and Gastrointestinal Tumor, The First Affiliated Hospital of Shandong First Medical University & Shandong Provincial Qianfoshan Hospital, Jinan, China; ^3^ Department of General Surgery, The First Affiliated Hospital of Shandong First Medical University & Shandong Provincial Qianfoshan Hospital, Jinan, China

**Keywords:** Wernicke’s encephalopathy, gastric cancer, thiamine, parenteral nutrition, psychiatric comorbidity

## Abstract

Wernicke’s encephalopathy (WE), a neuropsychiatric emergency caused by thiamine deficiency, is increasingly recognized in nonalcoholic populations. We present a 64-year-old male with pyloric obstruction from gastric cancer (stage IIIA) who developed WE 20 days after gastrectomy. Prolonged thiamine-free total parenteral nutrition (TPN), delayed enteral intake, and cancer-related hypermetabolism jointly precipitated a thiamine deficiency. Undisclosed psychiatric comorbidity exacerbated diagnostic challenges and potential risk. Despite initial diagnostic challenges, timely neurological assessment and urgent brain MRI confirmed the diagnosis on the day of readmission. Immediate thiamine supplementation led to full neurological recovery. At six-month follow-up, the patient remained neurologically intact with structured dietary and psychological counseling, ensuring sustained psychiatric stability during adjuvant chemotherapy. This novel case of WE following radical gastrectomy, prolonged TPN, and in the context of gastric cancer with pyloric obstruction and undisclosed psychiatric comorbidity, underscores the necessity of multidisciplinary collaboration to optimize perioperative nutritional and psychosocial management in high-risk oncological surgical populations.

## 1 Introduction

Wernicke’s encephalopathy (WE), an acute neuropsychiatric disorder caused by thiamine (vitamin B_1_) deficiency, was first described by Carl Wernicke in 1881 (1). The classic manifestation includes a triad of oculomotor abnormalities (nystagmus or ophthalmoplegia), cognitive impairment, and gait ataxia. Nonalcoholic patients often lack the classic triad. However, the complete triad appears in merely 10% of cases, contributing to frequent underdiagnosis in clinical practice (2). WE can present with atypical symptoms, leading to delayed diagnosis and treatment. While WE was initially recognized as being associated with chronic alcoholism, epidemiological studies indicate that nearly 50% of WE cases occur in nonalcoholic populations, particularly those with distinct clinical characteristics and neuroimaging features (3). The European Federation of Neurological Societies (EFNS) 2010 diagnostic criteria build upon Caine’s diagnostic framework proposed in 1997, emphasize nutritional deficiencies, oculomotor signs, cerebellar dysfunction, and altered mentation (4, 5). The estimated prevalence ranges from 0.8% to 2.8%, with autopsy studies indicating that approximately 80% of cases remain undiagnosed ante-mortem (6). Malignancy and major gastrointestinal surgery represent significant etiological factors in nonalcoholic WE, accounting for 18% and 16.8% of cases respectively (7, 8). In oncological patients, chronic malnutrition, chemotherapy-induced vomiting, and cancer-related hypermetabolism collectively create multiple risk factors for nutrient deficiency. Gastrointestinal surgeries exacerbate this vulnerability by disrupting anatomical structures for nutrient absorption. Psychiatric comorbidities further amplify nutritional risks through maladaptive behaviors and social disengagement. For surgical patients with undisclosed psychiatric conditions, this biological susceptibility interacts with metabolic stress and post-gastrectomy malabsorption.

We report a novel case of postoperative WE in an advanced gastric cancer patient with pyloric obstruction. The patient’s undisclosed and untreated obsessive-compulsive disorder (OCD) and generalized anxiety disorder (GAD) posed distinct diagnostic challenges during clinical evaluation. This case underscores the prognostic significance of early risk stratification and the necessity of multidisciplinary perioperative management integrating nutritional and psychosocial evaluations, as detailed in the subsequent analysis.

## 2 Case report

A 64-year-old male (Body Mass Index, BMI 25.8 kg/m²) with controlled hypertension and gout presented with 1-month history of progressive postprandial epigastric distension, non-bilious vomiting, and reflux. He denied alcohol use or prior cerebrovascular events. Persistent gastric food residue necessitated three gastroscopies: one performed at an external institution and two at our hospital, leading to prolonged inpatient care for diagnostic evaluation in the Department of Gastroenterology. During this period, nasogastric decompression, nil per os (NPO) status, and total parenteral nutrition (TPN) were implemented for 10 days. The patient was referred to the Department of General Surgery after the confirmation of biopsy-proven well-differentiated intramucosal adenocarcinoma with pyloric obstruction.

Preoperatively, the interventions (nasogastric decompression, NPO status, and TPN) were continued for an additional 2 days, totaling 12 days of preoperative thiamine-free management. Preoperative risk stratification included standard medical evaluations. However, formal psychosocial screening using standardized tools was not documented during this initial assessment, and the patient did not disclose a history of psychiatric conditions at that time. Bowel preparation including mechanical lavage preceded the laparoscopic radical distal gastrectomy with Billroth II reconstruction. Histopathology revealed moderately-poorly differentiated adenocarcinoma (pT4a N2 M0, stage IIIA).

Postoperatively, delayed bowel recovery and postsurgical gastroparesis syndrome (PGS) required prolonged nasogastric decompression (discontinued on POD 7) and graded enteral intake (liquid diet on POD 7, semiliquid by POD 11). Abdominal drainage was removed on POD 10, and the patient was discharged with stable wound healing.

On POD 20, he was readmitted with 3-day progressive neurological decline: dizziness, gait instability, dysarthria, anterograde amnesia, and food refusal. Significantly, collateral history obtained from family members disclosed a 5-year history of unreported and untreated OCD characterized by pathological checking compulsions, along with inadequately managed GAD, neither of which had been documented in the initial admission records.

**Figure 1:**
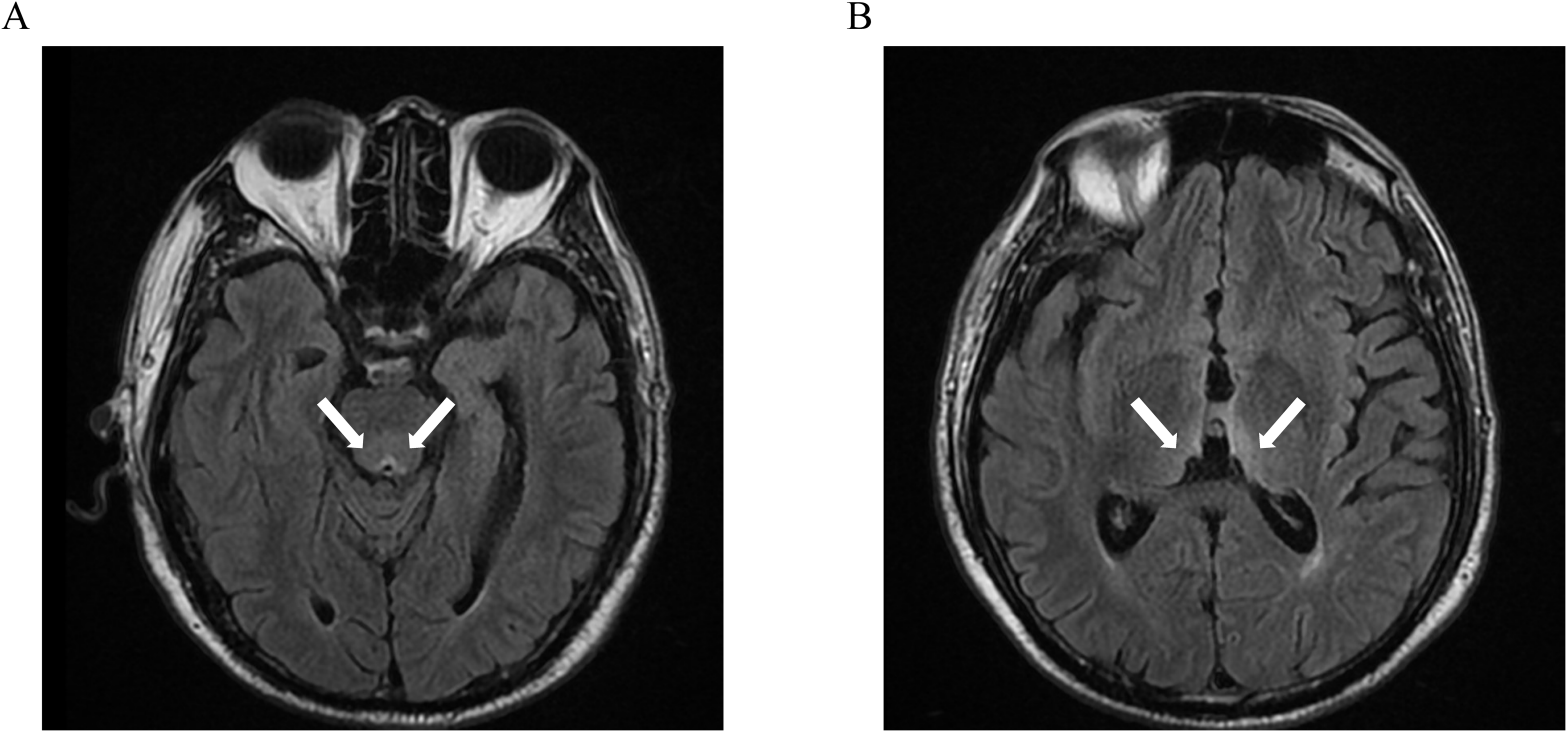
Symmetric T2-FLAIR hyperintense lesions involving the periaqueductal region **(A)**, bilateral thalami, and the margins of the third ventricle **(B)**.

Neurological examination showed right gaze palsy, horizontal nystagmus, generalized hyporeflexia, and asymmetric limb weakness (left upper limb 4/5, bilateral lower limbs 3+/5). Laboratory tests indicated hypokalemia (3.04 mmol/L) and mild hyponatremia (130.3 mmol/L). Urgent brain MRI demonstrated bilateral thalamic, periaqueductal, and third ventricular T2-FLAIR hyperintensities (**Figure 1**).

Following neurological consultation and exclusion of differential diagnoses of altered mental status and neurological deficits, the timely diagnosis of WE was confirmed based on clinical presentation, risk factors, and urgent, highly suggestive brain MRI findings obtained on the day of readmission. Immediate intramuscular thiamine (100 mg thrice daily) and electrolyte correction were initiated. Neurological deficits resolved completely within 7 days.

**Figure 2:**
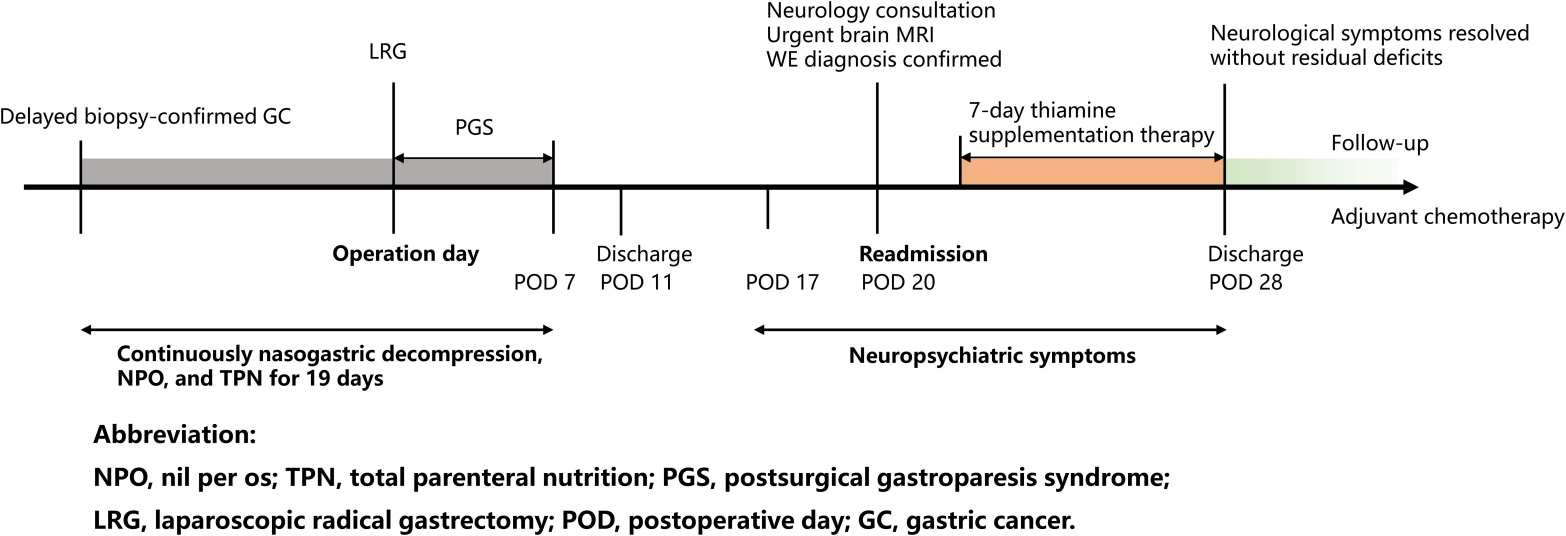
Timeline of the patient’s disease development.

At 6-month follow-up, structured dietary support including oral thiamine supplementation and psychological support ensured neurological integrity and psychiatric stability without neurological residual deficits during adjuvant chemotherapy (4). Structured dietary support included regular monitoring of laboratory tests and tailored nutritional counseling to address the risk of post-gastrectomy malabsorption. Psychological support comprised monthly sessions with a multidisciplinary team to manage psychiatric comorbidity and ensure adherence to long-term nutritional protocols (**Figure 2**).

## 3 Discussion

WE is caused by cerebral thiamine deficiency, classically associated with alcoholism (90% of cases) but increasingly recognized in nonalcoholic populations (9). Thiamine, a water-soluble vitamin essential for carbohydrate metabolism and neuronal signaling, is rapidly depleted within 18 days without adequate intake in healthy adults (6). Thiamine deficiency triggers lactate-mediated cerebral acidosis and neuronal hyperexcitability (10, 11). Key mechanisms of deficiency include insufficient dietary intake (e.g., unbalanced diets or thiamine-free TPN), impaired absorption secondary to anatomical obstructions (e.g., pyloric obstruction, pyloric stenosis) or functional disorders (e.g., hyperemesis gravidarum), metabolic imbalances (e.g., excess carbohydrate load), and hypermetabolic states (e.g., during growth periods, strenuous physical activity, gestation, malignancy or infection) (12). Alcohol exacerbates these pathways but was absent in our case.

This case illustrates multifactorial WE pathogenesis in a patient after radical gastrectomy, emphasizing the critical interplay between surgical interventions, nutritional management, cancer biology, and psychosocial factors. Radical gastrectomy further compounded these risks through inadequate dietary intake, impaired digestion with subsequent malabsorption, and reduced gastric acid secretion critical for thiamine release (6). Chronic pyloric obstruction caused prolonged vomiting and malnutrition, compounded by undiagnosed psychiatric comorbidities that likely worsened food refusal and by cancer-related hypermetabolism that may accelerate thiamine depletion. Preoperative delayed diagnostic gastroscopy and PGS necessitated prolonged thiamine-free TPN, while subclinical anxiety may have further compromised gastrointestinal recovery through psychosomatic interactions, potentially contributing to PGS and food refusal that prolonged TPN (13). Postoperative antibiotics and acid suppressants may have additionally impaired thiamine absorption (14). Despite the absence of neoadjuvant therapy in this case, the advanced disease requires heightened vigilance for metabolic risks during adjuvant chemotherapy (15).

The diagnosis of WE requires integration of clinical context, neuroimaging, and laboratory parameters (16). Clinical presentation, particularly oculomotor abnormalities like nystagmus, is a key diagnostic feature and often precedes neuroimaging changes. Recent studies report WE-confirmed nystagmus even with normal MRI (17). MRI serves as the diagnostic imaging standard for WE, demonstrating 53% sensitivity and 93% specificity in confirmation of diagnosis (18). Characteristic T2/FLAIR hyperintensities with variable contrast enhancement typically involve the periventricular regions of the thalamus, hypothalamus, mammillary bodies, periaqueductal region, and floor of the fourth ventricular regions (19). In this case, ocular abnormalities and cognitive deficits may be misattributed to undisclosed psychiatric comorbidities, whereas overlapping postoperative conditions often obscure neurological signs, delaying diagnosis. Urgent brain MRI combined with multidisciplinary neurological evaluation enabled prompt diagnostic confirmation.

To ensure diagnostic rigor, alternative etiologies were systematically excluded. Hepatic encephalopathy was deemed unlikely due to the absence of a history of liver disease or neoadjuvant therapy-related liver injury, as well as normal liver function tests (20, 21). While mild hyponatremia was present, the prominent ocular and cerebellar manifestations strongly favored WE over hyponatremia-related delirium. Besides, metabolic encephalopathy from other causes (e.g., uremic encephalopathy, hypoglycemia) was excluded by normal renal function, glucose levels, and absence of systemic metabolic derangements beyond thiamine deficiency (22). It is important to note that direct measurement of serum thiamine levels was not routinely available at our institution at the time of presentation, despite the availability of assays for other vitamins like vitamin B_12_, vitamin A, vitamin E, et al. Although serum thiamine levels were not measured prior to treatment in this emergency, such measurement, when feasible, can be valuable for documentation and to differentiate WE from other causes of encephalopathy (e.g., central nervous system metastases, paraneoplastic syndromes) if the patient does not respond to thiamine therapy or if the diagnosis remains uncertain (23–25). The temporal relationship between thiamine depletion and symptom onset further strengthened the causal link to thiamine deficiency (6).

While MRI is a valuable diagnostic tool, empirical thiamine treatment should be initiated immediately based on clinical suspicion, especially in high-risk patients presenting with characteristic signs, without waiting for imaging or laboratory confirmation (26).Prompt parenteral thiamine is critical, though optimal dosing regimens lack consensus (27). Current guidelines from EFNS recommend intravenous thiamine administration at 200 mg thrice daily (4). Our patient received 100 mg intramuscular thiamine administration thrice daily due to institutional constraints. This highlights the critical need to establish evidence-based regimens for dose optimization and treatment duration across clinical contexts.

The delayed recognition of psychiatric comorbidities in this case highlights systemic shortcomings in psychosocial assessment within oncological surgery. In Chinese oncology practice, it is not uncommon for physicians to discuss cancer diagnoses primarily with family members rather than directly with the patient. Many families prefer to withhold or partially disclose unfavorable information to protect the patient (28). In our case, the limited self-report and the protective communication style may have contributed to under detection of preoperative OCD and GAD. Verbal and written dietary guidance following standard post-gastrectomy home care protocols was provided to the patient after the initial discharge. The guidance included detailed instructions on nutrient-dense meal planning, small-frequent feeding schedules, and monitoring for malnutrition indicators (29). Despite these interventions, the documented food refusal and progressive neurological decline upon readmission strongly indicate inadequate nutritional intake during this period. Furthermore, while a compulsive personality might be expected to demonstrate compliance, untreated OCD and GAD in the context of severe illness, prolonged NPO and TPN, and cancer-related psychological stress can lead to maladaptive coping mechanisms, including significant anxiety surrounding food intake or outright food refusal, thereby compounding nutritional deficiencies. In this complex clinical scenario, the patient exhibited such maladaptive behaviors, as evidenced by the documented food refusal observed during readmission. This likely resulted from impaired adherence to the prescribed dietary regimen due to the individual’s psychiatric comorbidities. The case highlights how severe, unmanaged mental health conditions can paradoxically undermine essential nutritional intake, particularly when compounded by concurrent physical illness and surgical recovery.

Moreover, the protracted diagnostic process for pyloric obstruction which included three separate gastroscopies and several weeks of malnutrition may have precipitated further psychological deterioration, thereby diminishing the sensitivity of routine screening instruments. Cancer-related psychological adaptation is a complex, dynamic process (30). The combined metabolic and emotional burden of a cancer diagnosis creates a distinctive and complex context for the psychiatric evaluations.

Standardized tools like Nutritional Risk Screening-2002 (NRS-2002) and Malnutrition Universal Screening Tool (MUST) are essential for identifying patients at risk of malnutrition (31). However, our systematic review of nutritional support practices at our hospital revealed some gaps in water-soluble vitamin supplementation despite routine application of NRS-2002. These findings prompted us to implement preventive protocols, including mandatory thiamine supplementation in TPN regimens for high-risk patients and enhanced psychiatric screening in preoperative oncology assessments.

This case highlights the critical need for institutional-level reviews following adverse events to refine clinical pathways. For patients with gastric cancer, particularly those in advanced stages, standardized whole-course management should systematically integrate refined surgical techniques and precise with individualized perioperative adjuvant treatment strategies, while equally emphasizing nutritional surveillance and longitudinal psychosocial health monitoring (32). Future clinical practice requires the comprehensive integration of multidisciplinary team collaboration, while incorporating culturally attuned psychosocial assessment tools, such as structured family interviews and assessment tailored to the oncology setting, to ensure systematic identification of mental health needs and thereby improve overall treatment outcomes. Moreover, effective physician-patient communication and treatment adherence optimization are crucial to enhance adherence with personalized therapeutic, ultimately aiming to improve clinical outcomes and quality of life (33). By integrating these measures into institutional policy, we aim to reduce the incidence of similar adverse events and improve outcomes for patients with complex comorbidities.

## 4 Conclusion

This case of WE after radical gastrectomy underscores synergistic risks associated with cancer-related hypermetabolism, undisclosed psychiatric comorbidities, and delayed enteral nutrition. Early neuroimaging and interdisciplinary collaboration facilitated the timely reversal of neurological deficits, highlighting the importance of integrated nutritional and psychosocial monitoring in the comprehensive management of patients with gastric cancer.

## Data Availability

The raw data supporting the conclusions of this article will be made available by the authors, without undue reservation.
